# Relationship between Circulating and Tissue microRNAs in a Murine Model of Breast Cancer

**DOI:** 10.1371/journal.pone.0050459

**Published:** 2012-11-30

**Authors:** Peadar S. Waters, Ailbhe M. McDermott, Deirdre Wall, Helen M. Heneghan, Nicola Miller, John Newell, Michael J. Kerin, Roisin M. Dwyer

**Affiliations:** 1 Discipline of Surgery, School of Medicine, National University of Ireland, Galway, Ireland; 2 HRB Clinical Research Facility, National University of Galway, Galway, Ireland; 3 School of Mathematics, Statistics and Applied Mathematics, National University of Galway, Galway, Ireland; H. Lee Moffitt Cancer Center & Research Institute, United States of America

## Abstract

MiRNAs are key regulators of tumorigenesis that are aberrantly expressed in the circulation and tissue of patients with cancer. The aim of this study was to determine whether miRNA dysregulation in the circulation reflected similar changes in tumour tissue. Athymic nude mice (n = 20) received either a mammary fat pad (n = 8, MFP), or subcutaneous (n = 7, SC) injection of MDA-MB-231 cells. Controls received no tumour cells (n = 5). Tumour volume was monitored weekly and blood sampling performed at weeks 1, 3 and 6 following tumour induction (total n = 60). Animals were sacrificed at week 6 and tumour tissue (n = 15), lungs (n = 20) and enlarged lymph nodes (n = 3) harvested. MicroRNAs were extracted from all samples (n = 98) and relative expression quantified using RQ-PCR. *MiR-221* expression was significantly increased in tumour compared to healthy tissue (p<0.001). *MiR-10b* expression was significantly higher in MFP compared to SC tumours (p<0.05), with the highest levels detected in diseased lymph nodes (p<0.05). *MiR-10b* was undetectable in the circulation, with no significant change in circulating *miR-221* expression detected during disease progression. *MiR-195* and *miR-497* were significantly decreased in tumour tissue (p<0.05), and also in the circulation of animals 3 weeks following tumour induction (p<0.05). At both tissue and circulating level, a positive correlation was observed between *miR-497* and *miR-195* (r = 0.61, p<0.001; r = 0.41, p<0.01 respectively). This study highlights the distinct roles of miRNAs in circulation and tissue. It also implicates miRNAs in disease dissemination and progression, which may be important in systemic therapy and biomarker development.

## Introduction

It is currently recognised that breast cancer is a heterogeneous disease that comprises several distinct molecular subtypes [1]. Basal type breast cancer is a subtype characterised by a lack of protein expression of oestrogen receptor (ER) and progesterone receptor (PR) and the absence of HER2 protein over expression. It is associated with poor outcome compared to other subtypes due to its poor disease free survival in the post-operative setting, lack of targeted adjuvant hormonal treatment options and increased metastatic potential [2], [3]. Considering the incidence of breast cancer, sensitive and specific biomarkers for the detection of disease initiation and progression are crucial to increase early detection in patients with the disease. To date it has been reported that screening mammography has a sensitivity ranging from 62.9%–87% [4]. The use of CA15.3 as an adjunct in screening and prognostication is also limited since it is only raised in 10% of stage I and 20% stage II breast cancers [5], [6], [7]. Therefore the quest for a specific, sensitive and non invasive biomarker for the detection of breast cancer continues. The discovery of miRNAs as novel modulators of gene expression has resulted in extensive investigation into the ability of miRNAs to act as biomarkers of disease. First implicated to be relevant in disease biogenesis and clinical behaviour, these small regulatory RNA molecules modulate the activity of specific miRNA targets and therefore play a functional role in a wide range of disease processes [8]. A fundamental understanding of miRNA interactions and relationships is imperative prior to clinical translation.

The detection of miRNAs in both circulation and tumour tissues has led to the search for miRNAs to predict presence of cancer and indicate its overall prognosis. In 2005, it was reported that a miRNA signature characterised human breast cancer subtypes suggesting their involvement in breast cancer tumourigenesis [9]. Further analysis of miRNA profiling in breast cancer tissue has shown multiple miRNAs to be aberrantly expressed and serve as oncogenic agents or tumour suppressors [10]. Early studies have implicated miR-21, 155 and 206 to be over-expressed in tumours whereas miR-125b and miR-145 were found to be downregulated [11]. The first report of circulating miRNAs in patients with diffuse large B-cell lymphoma documented elevated serum levels of *miR-21* [12]. Subsequently, circulating miRNAs have been shown to be relatively stable and detectable in both serum and plasma [13]. Breast cancer specific blood-based miRNAs such as *miR-195* have been shown to be upregulated in cancer patients compared to controls and return to normal levels post tumour excision [14]. Moreover studies have further characterised specific circulating miRNAs such as miR-155 to be aberrantly expressed in certain subtypes of breast cancer [15]. These seminal findings have paved the way for detection and quantification of circulating miRNAs and the elucidation of their potential role as novel non-invasive biomarkers of cancer. Despite these findings, unanswered questions still remain surrounding the ability of circulating miRNAs to reflect changes in tumour tissue with little data available regarding the relationship between the two.

It is reported that *miR-10b* is down-regulated in breast tumour tissue but over-expressed in patients with metastatic disease burden [16], [17], [18], [19]. *MiR-221* over-expression in tissue has been implicated in liver tumourigenesis and as a key modulator of aggressive prostate cancer [20], [21]. *MiR-195* and *497* have been shown to be significantly down regulated in breast tumour tissue [22]. In contrast circulating *miR-195* has been reported to be significantly upregulated in patients with breast cancer [23]. Elevated circulating miR-221 has been associated with resistance to neoadjuvant chemotherapy in breast cancer [24]. The source of circulating miRNAs is also currently a topic of much debate. Two hypotheses proposed by *Slack et al* postulate that miRNAs are present in the circulation due to tumour cell death and cell lyses. Alternatively they propose that tumour cells release miRNAs into the surrounding microenvironment and enter the circulation during angiogenesis [25]. Studies also propose their presence in the circulation via exosomal release from cells [26], [27], [28]. MiRNA changes in circulation may also be related and altered due to the host immune response or inflammatory reactions rather than intrinsic changes within the tumour.

To date there has been little evidence investigating the relationship between circulating & tumour tissue miRNA expression. The primary aim of this study was to investigate if miRNA dysregulation in the circulation reflected similar changes in tumour tissue in a murine model of breast cancer. Concurrently this model facilitates tumour induction and interval blood sampling in a controlled environment to establish if miRNA release in the circulation reflects tumour progression. The use of athymic mice models also allows accurate analysis of miRNA expression in blood and tissue in the absence of any significant host immune response to tumour induction.

## Materials and Methods

### Cell Culture

The breast cancer cell line MDA-MB-231 was obtained from the American Type Culture Collection (ATCC) and cultured in Leibowitz-15 (L-15) supplemented with 10% fetal bovine serum (FBS), 100 IU/ml and Penicillin G (2 units/mL)/Streptomycin sulphate (100 mg/mL). Cells were cultured in a humidified atmosphere at 37°C and 5% CO_2_ with a media change twice weekly and passage every 7 days. MDA-MB-231 cells were trypsinised into single cell suspensions, counted with a Nucleocounter® (Chemometic), and centrifuged. 2×10^5^ or 4×10^5^cells were re-suspended in 0.2 mls 50% Matrigel medium at 4°C for injection as described below.

### In Vivo Model

**Figure 1:**
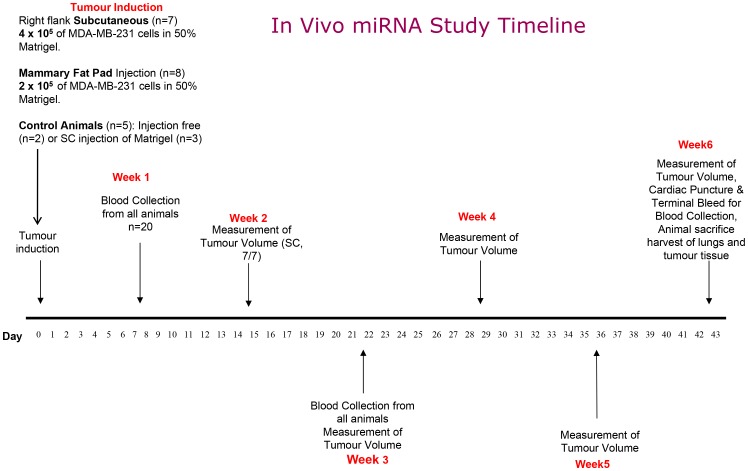
In Vivo miRNA study timeline. This study comprises of 20 athymic nude mice of which 15 were tumour bearing (MFP = 8 and SC = 7) and 5 were controls. Blood sampling was carried out from all animals at weeks 1, 3, and 6 as highlighted with red arrows. Tumour volume and weight measurements were undertaken at a weekly basis for the 6 week duration of the project. Animals were sacrificed at week 6 following tumour induction and all primary tumours (n = 15), lungs (n = 20) and lymph nodes (n = 3) were harvested for analysis and a terminal bleed via cardiac puncture was performed.

Twenty Female athymic nude mice (Harlan Sprague-Dawley, Indianapolis, IN) received a mammary fat pad (MFP, n = 8) or subcutaneous right flank (SC, n = 7) injection of 2×10^5^ or 4×10^5^ respectively of MDA-MB-231 cells in 0.2 ml 50% Matrigel medium. Control mice received no injection of tumour cells (n = 5). Blood sampling was carried out on all animals from the lateral tail vein at weeks 1, 3 and 6 following induction (total n = 60, Fig. 1). Blood was stored at 4°C in 2 ml EDTA tubes. Mice were weighed weekly and all tumour volumes were measured weekly using callipers, and estimated according to the formula: (mm)^3^ = L×W×D×0.52. At week 6 all mice were sacrificed by CO_2_ inhalation. A terminal bleed by pericardiocentesis was carried out and tumour tissue and lungs were harvested from all mice for analysis by RQ-PCR. Three mice were noted to have enlarged lymph nodes which were also harvested. All tissues were immediately snap frozen in liquid nitrogen and stored at −80°C until required. All animal experiments were licensed and carried out following ethical approval by, and according to the guidelines of, the National University of Ireland Galway Animal Care Research Ethics Committee (Permit Number: B100/3751).

### RNA Isolation from Whole Blood and Tissue

Total RNA was extracted from 50 µL of blood using TRI Reagent BD technique (Molecular Research Centre, Inc.). [23] Murine tissue (0.5–2 mg) was homogenized using a bench-top homogenizer (Polytron® PT1600E; Kinematica AG, Littau-Luzem, Switzerland) in 1 ml TRIzol Reagent (Invitrogen, Carlsbad, CA). Total RNA was isolated from homogenized tissue using the RNeasy® Tissue Mini Kit (Qiagen) according to the manufacturer’s instructions. The miRNA concentration and purity were assessed by NanoDrop™ 1000 spectrophotometry (Nanodrop Technologies, Wilmington, DE, USA) and Agilent Bioanalyser (Agilent technologies, Germany).

### Analysis of miRNA Expression

RQ-PCR quantification of miRNA expression was performed using TaqMan® MicroRNA Assays (Applied Biosystems, Foster City, CA, USA) according to the manufacturer’s protocol. MiRNA (100 ng) was reverse-transcribed using the MultiScribe™-based High-Capacity cDNA Archive kit (Applied Biosystems). RT-negative controls were included in each batch of reactions. PCR reactions were carried out in final volumes of 10 µl using a 7900 HT Fast Real-Time PCR System (Applied Biosystems). Briefly, reactions consisted of 0.7 µl cDNA, 1× TaqMan® Universal PCR Master Mix, 0.2 µM TaqMan® primer–probe mix (Applied Biosystems). Reactions were initiated with 10-minute incubation at 95°C followed by 40 cycles of 95°C for 15 seconds and 60°C for 60 seconds. The expression of a panel of breast cancer associated miRNAs (*miR-10b, miR-221, miR-195* and *miR-497*) was examined on the basis of their reported relevance [29], [30]. *miRNA-16* and *let-7a* were used as endogenous controls to standardize miRNA expression for blood and tissue respectively [29]. Triplicate samples, validated endogenous controls, and interassay controls were used throughout. MiRNA expression levels were calculated and the threshold standard deviation for intra-assay and inter-assay replicates was 0.3. The relative quantity of miRNA expression was calculated using the comparative cycle threshold (ΔΔCt) method [31]. The geometric mean of the cycle threshold value of the endogenous control genes was used to normalize the data, and the lowest expressed sample was used as a calibrator.

### Statistical Analysis

Due to the magnitude and range of relative miRNA expression levels observed, a log transformation was applied to each expression level. Summary statistics and graphical techniques were used to summarise and compare the change in mean log expression level for each response variable (i.e. miR-497, miR-195 and miR-221) between the groups (i.e. Control, MFP and SC) and across time (i.e. Weeks 1, 3 and 6).

A linear mixed model for a longitudinal continuous response was used to compare the level in each response across time and between groups with a random effect term included to model the correlation within each mouse across time. [32] The need for two way interactions between group and time was investigated for each response. Appropriate model checking, based on residual plots at the subject and cluster level, was performed for all mixed models and the significance level for all analyses was set at the 5% level. All analyses were carried out using Minitab 16 and R (2.14.2). The 2-sample t test was used for all 2 sample comparisons and ANOVA, followed by Tukey HSD post hoc test for inter-sample comparisons.

## Results

### Detection of miRNAs in Tumour Tissue

**Figure 2:**
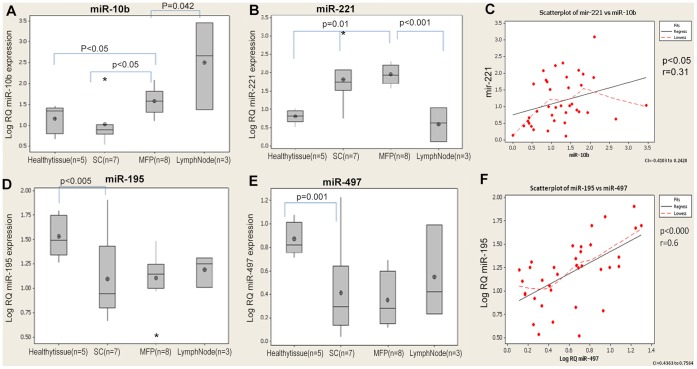
Relative expression of MiRNAs in tumour (n = 15), lymph nodes (n = 3) and healthy tissue (n = 5). (**A**) miR-10b expression. (**B**) miR-221 expression. (**C**) Positive correlation between miR-10b and miR-221 across all tissues. (**D**) MiR- 195 expression. (**E**) miR-497 expression. (**F**) Positive correlation between miR-195 and miR-497 expression across all tissues.

Subcutaneous tumour volume was measured using callipers on a weekly basis from week 2 to week 6 of tumourigenesis (Figure S1). MFP tumours could not be measured accurately using this method due to their shape, location and invasion of the local surrounding chest wall. At necropsy however all tumour tissue was harvested and weighed for accurate measurement of final tumour growth (Figure S2). The rate of tumour growth was significantly higher in MFP (mean±SEM, 194±33 mg) compared to SC (111±20 mg) tumours (p<0.05, Figure S2). MFP tumours were more advanced and macroscopically invaded more surrounding tissues compared to SC tumours. MFP tumours also had a higher incidence of lymph node metastases. *MiR-16* and *let-7a* expression was stable across all tissue samples, and the average of both values was used as an endogenous control (C_T_ range: 21–25 across all samples, Figure S3A). Prior to in vivo inoculation, expression of *miR-10, miR-221, miR-195 and miR-497* was investigated in the cultured MDA-MB-231 cell line. RNA was extracted from cultured MDA-MB-231 cells, reverse transcribed and RQ-PCR carried out targeting *miR-195, miR-497, miR-221*, and *miR-10b*. Relatively robust expression of miR-195 and miR-221 was detected in the cultured cells, while miR-497 expression was significantly lower. MiR-10b expression was not detectable in the cells prior to inoculation in vivo. Tumour induction site influenced expression of *miR-10b* with significantly higher levels expressed in MFP tumours compared to SC tumour (p<0.05, Figure 2A), with highest detected in malignant lymph nodes (n = 3, p<0.05, Figure 2A). *Mir-221* expression was upregulated in both MFP and SC tumour tissue compared to controls (p<0.01), however returned to basal levels in lymph nodes (p<0.001, figure 2B), with no significant difference in expression between healthy tissue and diseased lymph nodes. A significant positive correlation was observed between miR*-10b* and *miR-221* across all tissues examined (r = 0.31, p<0.05, Figure 2C).

The expression levels of *miR-195* and *miR-497* in MFP and SC cancer tissues were significantly decreased when compared to healthy tissue (p<0.05, p<0.001, Figure 2D, E) respectively. The levels of both miRs were not significantly altered between lymph node and tumour tissue (p>0.05). There was a significant positive correlation between *miR-497* and *miR-195* detected in all tissues examined of the murine model (r = 0.61, p<0.001, Figure 2F).

### Analysis of Circulating miRNAs in Healthy versus Tumour-bearing Animals

**Figure 3:**
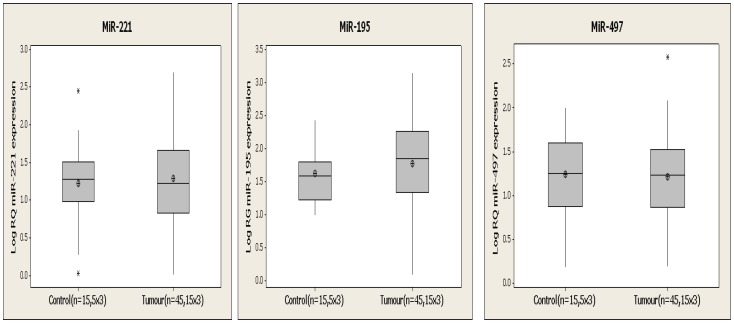
Circulating miRNA expression in tumour bearing (n = 45, 15×3) and healthy animals (n = 15, 5×3). At termination of this study, 6 weeks following tumour induction no significant difference was observed between circulating miR-221 (**A**), miR-195 (**B**) and miR-497 (**C**) in tumour bearing animals compared to healthy controls.

*MiR-10b* was not detected in the circulation of any samples in this study (n = 60). *MiR-16* expression in the circulation was stable across all 60 samples (C_T_ range: 27–30 across all samples, Figure S3B) and was used as an endogenous control. *MiR-221*, *miR-195*, and *miR-497* were detected in all samples and expression was found to remain unchanged in healthy controls throughout the 6 week duration of the study. At 6 weeks following tumour induction there was no significant difference in *miR-221* (Figure 3A), *miR-195* (Figure 3B), or *miR-497* (Figure 3C) expression at a circulating level in tumour bearing animals (n = 15) compared to healthy controls (n = 5). Furthermore there was no significant relationship between the expression of circulating *miR-221*, *miR-195 and miR-497* at week 6 and final tumour volume detected.

### Detection of Circulating miRNAs during Tumour Progression

**Figure 4:**
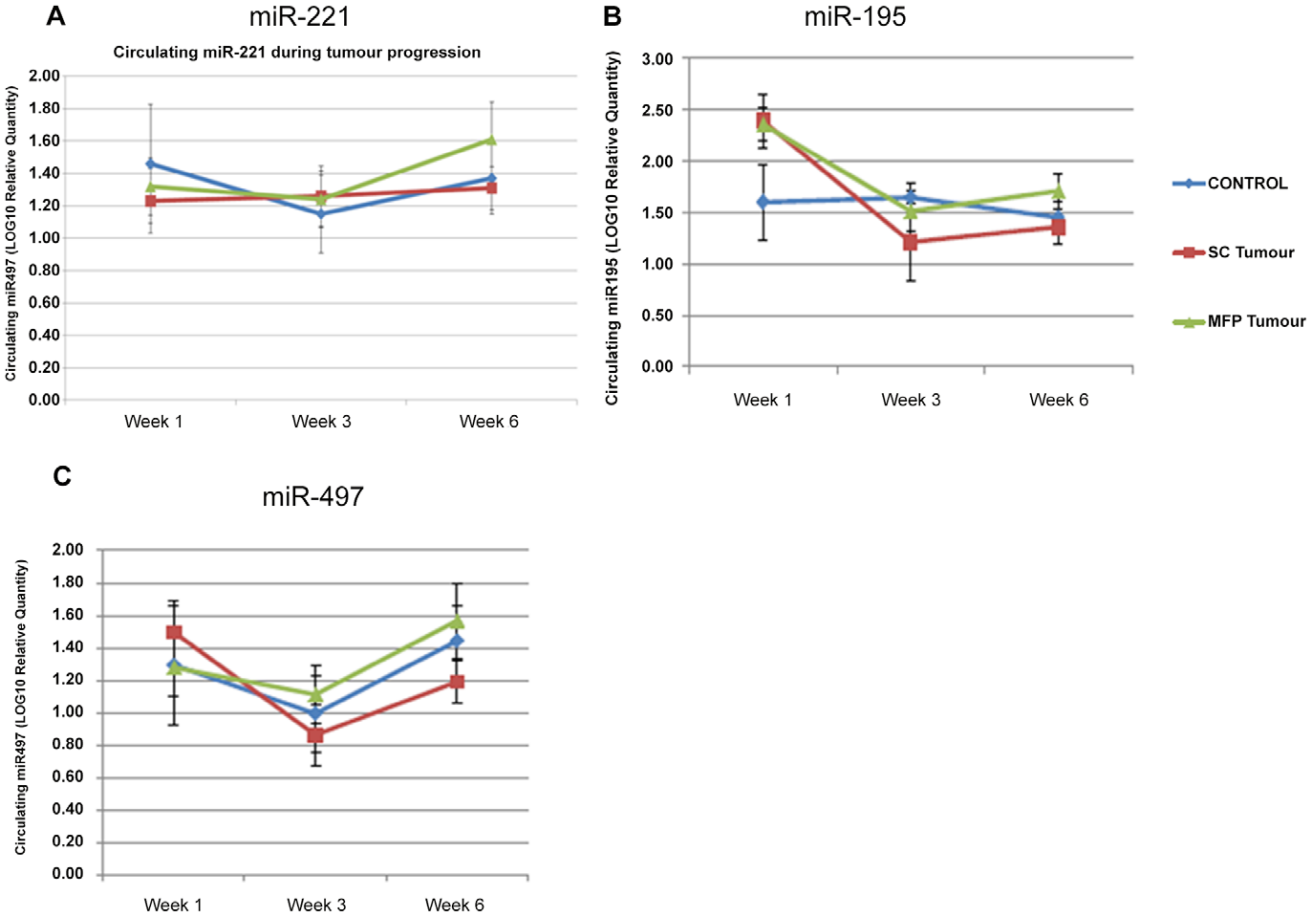
Circulating miRNA expression during tumour progression over 6 weeks. Relative levels of circulating miRNAs were quantified 1, 3, and 6 weeks following tumour induction to investigate the relationship with disease progression (n = 20 at each time point, total n = 60) Circulating miR-221 was no significantly altered during the 6 week study (**A**)**.** Circulating *miR-195* and *miR-497* was significantly decreased at week 3 following tumour induction (**B, C**). *MiR-195* and *miR-497* expression was observed to increase again at week 6, however this was not significant.

**Figure 5:**
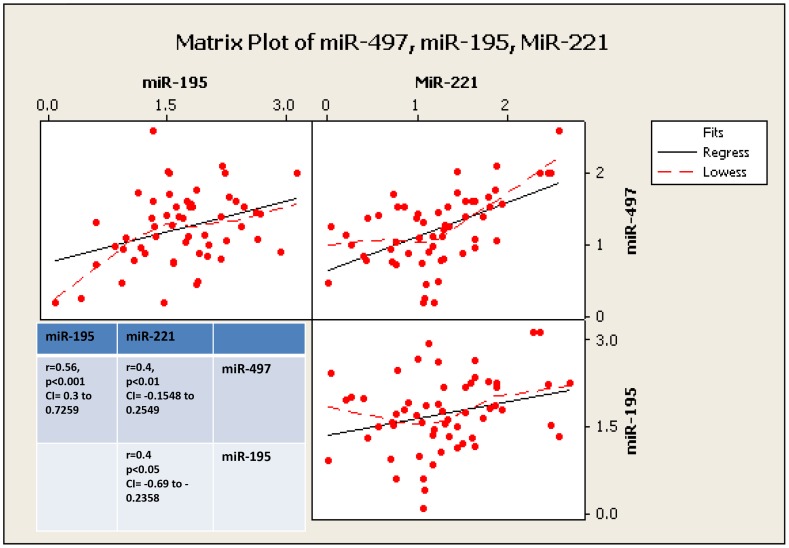
Investigation of relationships between circulating miRNA. Further significant positive correlations were observed between circulating *miR-195* and miR-497, circulating miR-195 and miR-221, and between *miR-497* and *miR-221*.

To analyse the potential impact of tumourigenesis on circulating miRNAs at specific time points, the levels of miRNA expression in the circulation of each animal was determined at week 1, 3 and 6 following induction (n = 20 samples at each timepoint, total n = 60). Plots of the change in mean log expression for each response variable across time and between groups are given in Figure 4. In the case of *miR-221*, following an initial decrease between week 1 and 3, there was a trend towards increased levels by week 6 in animals bearing MFP tumours, although overall expression was not significantly altered from tumour induction to termination time at week 6 (Figure 4A). The mean log *miR-195* expression at Week 1 is higher compared to Week 3 (p<0.001) and to Week 6 (p = 0.002) with no evidence of a significant difference between Weeks 3 and 6 (p = 0.98). There was evidence of a possible interaction between group and time where mean log *miR-195* expression for the SC group compared to the controls at week 3 compared to week 1 (p = 0.02, Figure 4). There was evidence of a significant difference in mean log *miR-497* expression across time where the level was significantly lower at Week 3 compared to Week 1 (p = 0.049) and Week 6 (p = 0.05, Figure 4). There was no evidence of a significant difference in mean log *miR-497* between the three groups. Across all blood samples at each time point (total n = 60), a significant positive correlation was detected between *miR-195 & miR-497* in the circulation (r = 0.56, p<0.001, Figure 5). Further significant correlations were detected between *miR-221* and *miR-497* (r = 0.4, p<0.001, Figure 5) and *miR-221* and *miR-195* in the circulation (r = 0.4, p<0.05, Figure 5).

## Discussion

In order to realise the true potential of miRNAs to become classified as biomarkers for disease detection and prognostication, a greater understanding of miRNA expression and release is required. As outlined in the introduction, four miRNAs were chosen that have previously been shown to be associated with breast cancer in the literature [16], [22], [33], [34], [35], [36]. MiRNAs have been shown to be dysregulated in tissue and circulation in multiple benign and malignant neurological, cardiovascular and gastro-intestinal conditions [37], [38], [39], [40], [41], [42]. With such findings, questions have been raised as to whether this is reflective of the disease itself or secondary to host response. The use of an athymic murine model of breast cancer in the current study reduced the potential for a masking effect of the host immune response. This however does not exclude the effect of the miRNAs (originating from the tumour) on the host. Concurrently these mice do not harbour other disease, which facilitates accurate measurement of miRNA expression during tumour progression.

In support of previous reports, this study has demonstrated that macroscopic disease burden was dependant on tumour induction site [43]. Implantation of MDA-MB-231 cells into orthotopic (anatomically appropriate) sites affected the tumourigenic and metastatic properties of the breast cancer cell line. In our experience MFP tumour weight and volumes were significantly higher compared to subcutis tumour despite the fact that only half the numbers of cells were used for MFP tumour induction. Moreover MFP tumours accounted for increased local invasion and increased propensity for metastases and lymph node involvement, as previously described. This metastatic potential of MFP tumours was further highlighted in the study by significantly elevated *miR-10b* expression relative to SC tumours. *MiR-10b* has previously been highlighted as a potential marker for disease progression and invasion [16], [44], [45], [46]. Multiple studies have also outlined the association of elevated *miR-10b* as an indicator of poor prognosis and survival prediction [46], [47]. Indeed therapeutic silencing of *miR-10b* is being investigated as an approach to block or reduce disease metastases. [17]. In the current study *MiR-10b* was significantly higher in MFP tumours compared with SC tumours. Moreover it was demonstrated that *miR-10b* expression was highest in all diseased lymph nodes compared to primary tumour and healthy tissue (p<0.05, p<0.01) respectively. The authors acknowledge the limitation of a small number of mice with nodal metastases (n = 3), however despite this the specificity of *miR-10b’s* role in tumour invasion in this study was further validated by the fact that this up-regulation did not hold true for *miR-195,497* and *221.* Interestingly, *miR-10b* was not detected in the circulation of any animal (healthy or tumour bearing) at any time point highlighting the importance of miR-10b at a tumour micro-environment level rather than in the circulation.

*Mir-221*, an oncogenic miRNA implicated in multiple endocrine cancers was also investigated. Previously studied and shown to be aberrantly expressed in sporadic ovarian carcinoma, prostate and thyroid carcinoma, increased miR-221 has also been attributed to tamoxifen resistance in breast cancer [48], [49], [50], [51]. Moreover, it has been reported that *miR-221* is involved in the promotion of an aggressive basal-like phenotype in breast cancer, functioning downstream of the RAS pathway and triggering epithelial-to-mesenchymal transition [52]. In this study *miR-221* was upregulated in tumour tissue of both MFP and SC tumours compared to healthy tissue. In contrast to *miR-10b* however, *miR-221* was not seen to be over-expressed in diseased lymph nodes compared to controls. *miR-221* has also been studied in the circulation of patients with breast cancer with elevated levels in plasma reported to be predictive of resistance to neoadjuvant chemotherapy. [24], [53]. Unlike *miR-10b*, *miR-221* was readily detectable in the circulation of both diseased and healthy animals. We have established that there was a significant positive correlation between *miR-10b* and miR*-221* in all tissues, which to our knowledge has not been previously documented. There was no significant difference between circulating *miR-221* in tumour bearing animals and healthy controls at termination of the study. Furthermore circulating miR-221 was not reflective of tumour burden. This was possibly due to the short termination time of the project, which we acknowledge to be a limitation of the current study. This warrants further investigation in the setting of prolonged tumourigenesis.

*MiR-195* and *miR-497* have been shown to both originate from the *miR-16* super family [54]. Both miRNAs have been studied in tissue and circulation of patients with breast cancer. The data presented shows that both miRNAs were down regulated in tumour tissues when compared to healthy controls. Similar finding have also been reported in a recent study examining *miR-195* and *miR-497* in both primary human breast tumours and various breast cancer cell lines, highlighting their inhibitory role in breast cancer [22]. *MiR-195* and *miR-497* were both detected in circulation of all animals however were not significantly altered in expression in tumour bearing animals compared to controls at termination of the study. When analysing miRNA release over the 6 week study it was observed that both miR-195 and miR-497 were significantly decreased at week 3 following tumour induction. Despite this however, expression levels began to increase again at week 6. This increase was not significant however with prolonged tumourigenesis and the incremental increases that were observed within the final 3 weeks of the study, this may have become significant and warrants further investigation. This study highlights the importance of time point of measurement of circulating miRNA, considering the significant change in pattern of these circulating miRs over a relatively short timescale.

This study also explored the possible relationship between circulating and tissue miRNAs in the same animals which has not yet been investigated. Circulating levels of *miR-195, miR- 497* and *miR-221* were analysed and correlated with tissue levels from the same animals. Although no direct relationship between circulating miRNAs levels and tumour burden was observed, a significant positive correlation was observed between *miR-497* and *miR-195* within tissue. This correlation was also observed within all blood samples. This highlights that in this instance, dysregulation of miRNAs discovered in the circulation were reflective of tumour tissue. However in tissue, miR-10b was seen to have a direct relationship with disease progression, but remained undetected in the circulation at all time points examined. This may be a feature of the particular cell line used in tumour establishment. However, considering the high levels detected in tumours together with the fact that the other three miRNAs were detected in circulation, this warrants further investigation. A potential relationship between *miR-497* and tumour burden was also observed in this study although it failed to reach significance (p = 0.07).

### Conclusion

This study highlights the importance of miRNAs in breast cancer, with each displaying distinct roles in circulation and tissue. While some were shown to have a potentially important role in the primary tumour microenvironment, others displayed interesting patterns in the circulation. Furthermore the importance of the time point of miRNA measurement in the circulation is depicted. Interestingly, relationships between miRNAs observed at the circulating level were also mirrored in tumour tissue. Finally miRNAs were implicated in local tumour progression and systemic disease dissemination, which may be invluable in systemic therapy and biomarker development.

## Supporting Information

Figure S1 **Tumour volume during tumour progression.** Subcutaneous tumours were observed to increase in volume from day 14 after tumour induction up to the 6 week termination stage.
(TIF)

Figure S2 **Final Tumour weight at week 6.** Tumour growth and final tumour weight was significantly higher in MFP (n = 8) (mean±SEM, 194±33 mg) compared to SC (111±20 mg) tumours (n = 7) (p<0.05).
(TIF)

Figure S3 **Individual CT value of the endogenous control for Tissue and Blood.** **(A)**. The average of miR-16 and let-7a was used as endogenous controls for tissue samples and found to have a C_T_ range of a 21–25 across all 38 tissue samples included in this study. **(B)** MiR-16 was employed as an endogenous control for blood samples and was found to be within 3 CT values (C_T_ range: 27–30) across all 60 blood samples analysed.
(TIF)
