# Supplementary figures and images for: Relationship between Circulating and Tissue microRNAs in a Murine Model of Breast Cancer
Source: PLoS One. 2012 Nov 30;7(11):e50459. doi: 10.1371/journal.pone.0050459 (PMC3511577; doi:10.1371/journal.pone.0050459)

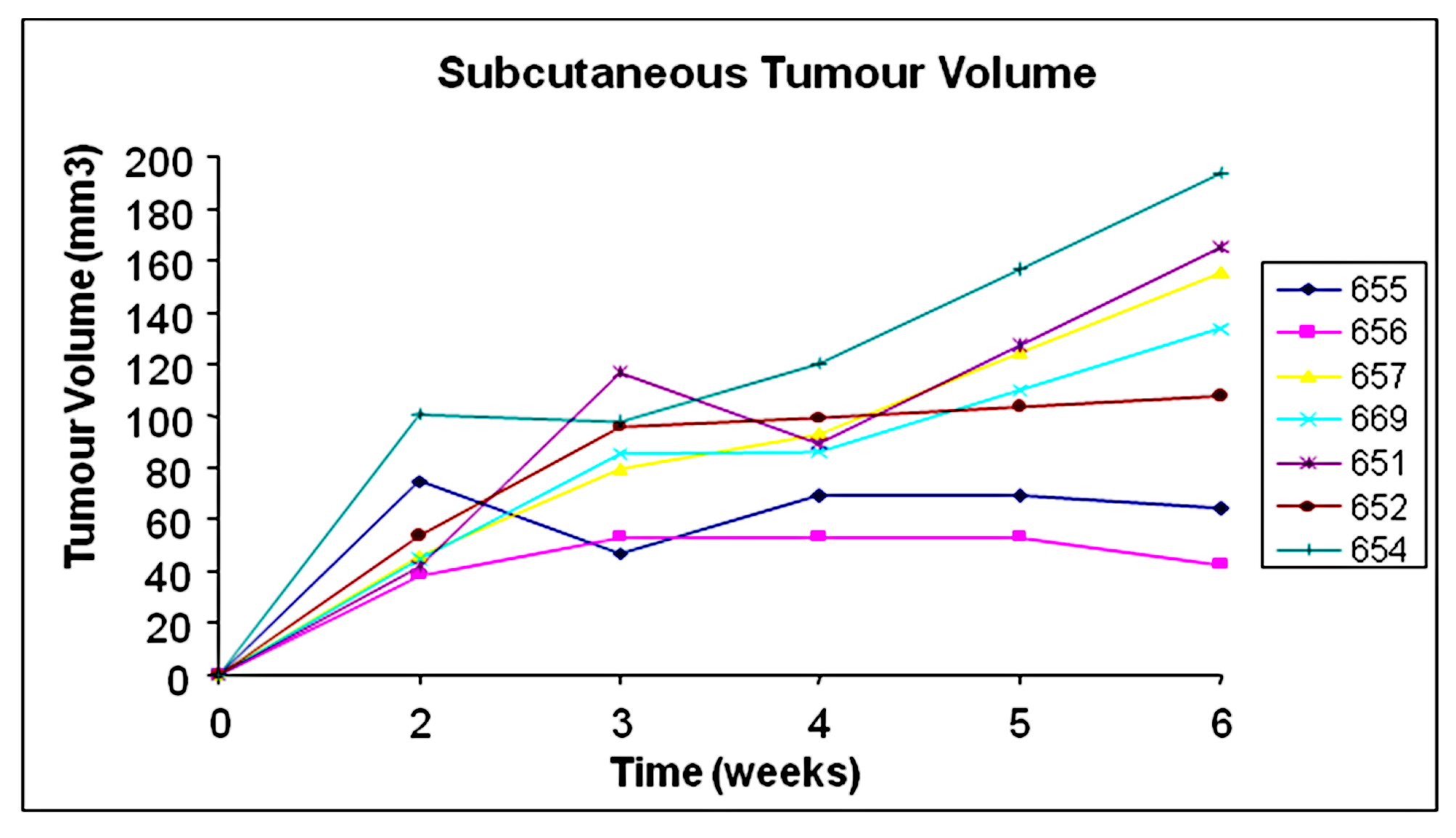

Supplement: Figure S1 — Tumour volume during tumour progression. Subcutaneous tumours were observed to increase in volume from day 14 after tumour induction up to the 6 week termination stage. (TIF) [file pone.0050459.s001.tif]

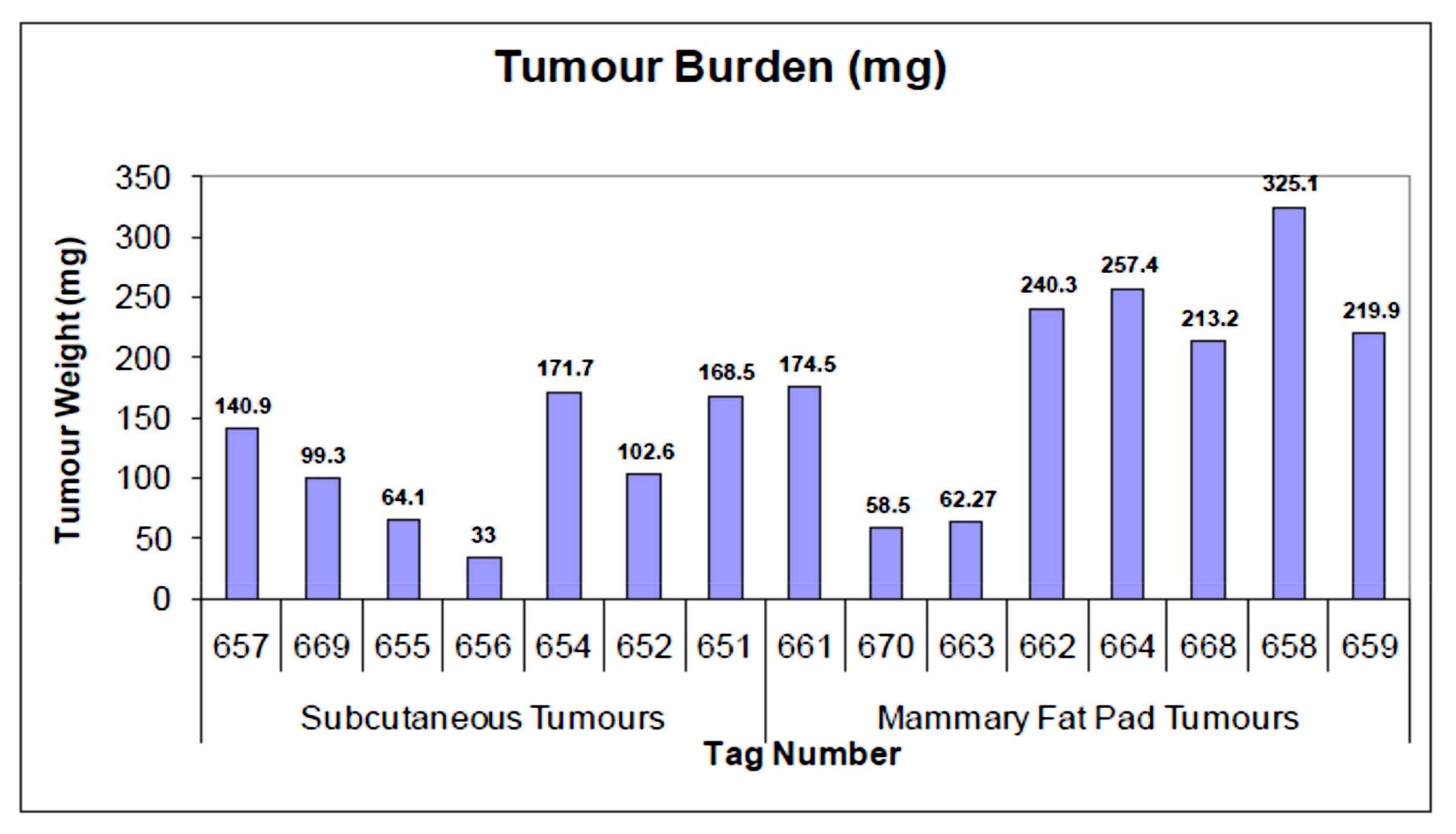

Supplement: Figure S2 — Final Tumour weight at week 6. Tumour growth and final tumour weight was significantly higher in MFP (n = 8) (mean±SEM, 194±33 mg) compared to SC (111±20 mg) tumours (n = 7) (p<0.05). (TIF) [file pone.0050459.s002.tif]

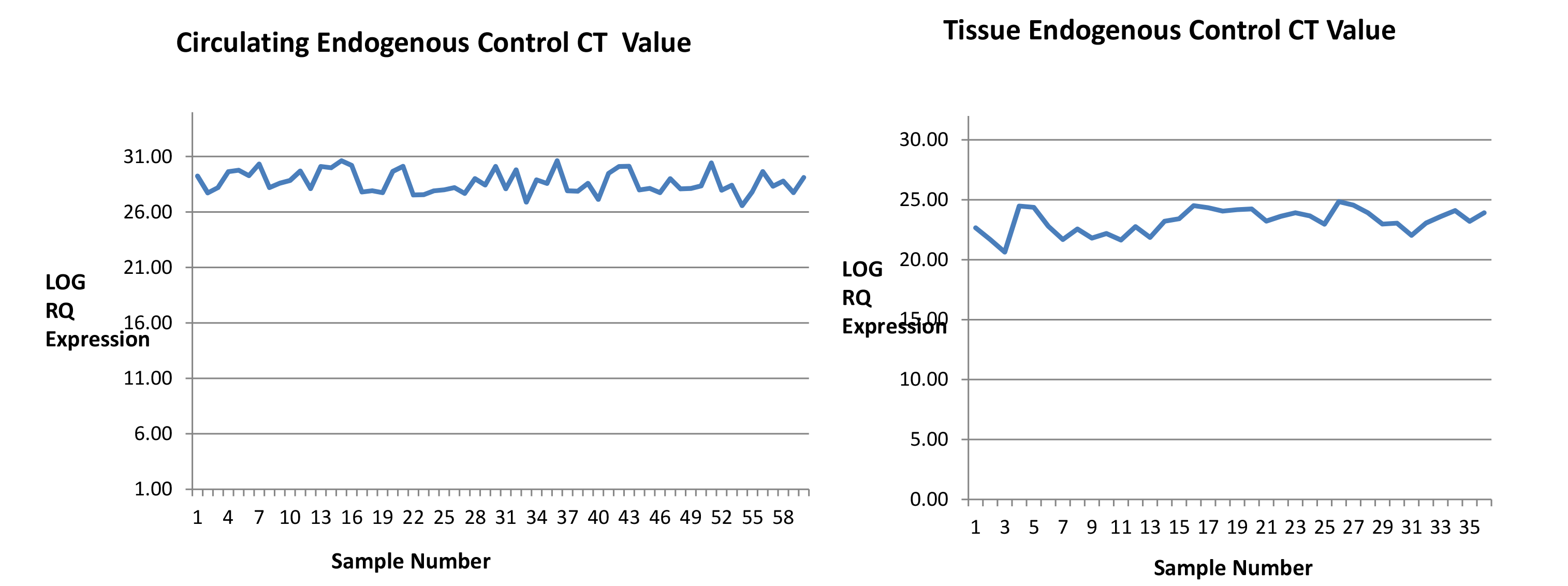

Supplement: Figure S3 — Individual CT value of the endogenous control for Tissue and Blood. (A). The average of miR-16 and let-7a was used as endogenous controls for tissue samples and found to have a CT range of a 21–25 across all 38 tissue samples included in this study. (B) MiR-16 was employed as an endogenous control for blood samples and was found to be within 3 CT values (CT range: 27–30) across all 60 blood samples analysed. (TIF) [file pone.0050459.s003.tif]
